# Numerical Validation of a Population Balance Model Describing Cement Paste Rheology

**DOI:** 10.3390/ma13051249

**Published:** 2020-03-10

**Authors:** Juan Pablo Gallo-Molina, Karel Lesage, Ingmar Nopens

**Affiliations:** 1 BIOMATH, Department of Data Analysis and Mathematical Modelling, Ghent University, Copure Links 653, 9000 Ghent, Belgium; 2 Magnel-Vandepitte Laboratory, Department of Structural Engineering and Building Materials, Ghent University, Technologiepark-Zwijnaarde 60, 9052 Ghent, Belgium

**Keywords:** cement pastes, rheology, population balance model

## Abstract

Rheology control is essential during the period in which cement and concrete pastes are encountered in the fresh state, due to the fact that it directly affects workability, initial placement and the structural performance of the hardened material. Optimizations of clinker formulations and reductions in cement-to-water ratios induced by economic and environmental considerations have a significant effect in rheology, which invokes the need for mechanistic models capable of describing the effect of multiple relevant phenomena on the observed paste flow. In this work, the population balance framework was implemented to develop a model able to relate the transient microstructural evolution of cement pastes under typical experimental conditions with its macroscopic rheological responses. Numerical details and performance are assessed and discussed. It was found that the model is capable of reproducing experimentally observed flow curves by using measured cluster size distribution information. It is also able to predict the complex rheological characteristics typically found in cement pastes. Furthermore, a spatially resolved scheme was proposed to investigate the nature of flow inside a parallel-plates rheometer geometry with the objective of assessing the ability of the model of qualitatively predicting experimentally observed behavior and to gain insight into the effect of possible secondary flows.

## 1. Introduction

Concrete is, by volume, the most produced material in the world, and it is a fundamental aspect of infrastructure development [1]. As such, its impact on both the global economy and the environment is enormous. Cement, the hydraulic binder responsible for the hardening of the material, is commonly thought to have been in use for around two millennia. However, a vast number of questions regarding the underlying mechanisms controlling various relevant processes still remain [1,2,3,4]. The reduction of cement content in concrete admixtures and the optimization of clinker formulations have been identified as viable and scalable pathways to reduce environmental impacts without compromising societal needs [1]. However, the application of these potential alternatives can impose challenges, among others, on the rheological behavior of cement and concrete pastes during the period they are encountered in the fresh state.

As stated by Chidiac and Mahmoodzadeh [3], appropriate rheological control is fundamental because workability is a basis for mixture design; flow behavior has a significant impact on the structural performance of the hardened material; and initial placement is directly dependent of rheological properties (especially viscosity and yield stress). Adequate description of rheology has, however, proven elusive due to the high compositional variability found within the family of cementitious materials, the presence of various significant phenomena and the different controlling mechanisms, which in turn depend on process conditions and composition [2]. The development of mechanistic models with predictive capabilities is thus challenging and yet of the utmost importance for the current engineering practice.

Nowadays, the rheological characterization of these systems is usually done via the Bingham model, which requires the plastic viscosity and yield stress to be known. In turn, appropriate modelling of those variables is of importance, as different processes are largely dominated by them (e.g., formwork filling is controlled by yield stress, while pumping is highly dependent on viscosity). Although the level of sophistication varies, most models currently used depend on a solids volume fraction as the main independent variable, and the use of empirical fitting is widespread [2,5,6,7,8,9,10,11,12]. While volume fraction is indeed a critical variable in the prediction of rheological parameters, significant departures between models and experimental data are usually found when no other variables are taken into account [8].

In addition to a solids volume fraction, a characteristic cluster size, whose selection is not trivial, is occasionally used in this field [2,13]. As could be expected, the choice of this characteristic size impacts directly the predicted properties and it is therefore a source of inaccuracies [2]. In actuality, however, the presence of a cluster size distribution (CSD) and its degree of polydispersity (which tends to be very large in cement pastes) play a very important role in the resulting rheological behavior [14,15,16]. Indeed, the presence of particles of different sizes induces effects such as self-lubrication, size-dependent differential clustering, depletion forces and structural differentiations caused by the lack of sphericity of the agglomerates [14,17,18,19,20,21,22].

In the case of cement pastes, the CSD is constantly evolving due to particle clustering, which initially is caused primarily by van der Waals and electrostatic interactions [2,4,8]. The implication is that even during the initial period of time after water addition, when rheology is not strongly related with the onset of hydration reactions [23,24], the transient changes in the size distribution ought to be kept into account. From the modelling perspective, the population balance framework is ideal to include CSD-related effects into a rheological model, as it allows the modeler to keep track of the time-dependent evolution of a system of particles, considering both their interactions with an environmental phase and themselves [25].

Population balance models (PBM) have been implemented in the past to bridge the gap between microstructure and rheological observables in complex suspensions [17,18,26]. Other methodologies, such as the weight-function-based models of Mwasame et al. [14] and Farris [27] or the recursive semi-empirical approach of Mendoza [20] have been proposed in the literature as well. In this work, we propose a PBM-based rheological model applied to fresh cement pastes. The presented formulation relates the transient microstructural evolution of cement suspensions with the change of relevant macroscopic variables, as opposed to typically used models. We discuss the ways in which agglomeration and breakage kernels can be used to describe the main hydrodynamic and interparticle interaction effects that play a role in rheology. Subsequently, we discuss the numerical performance of the model, and we analyze the complex rheological behavior emerging from the time-dependent evolution of the suspension microstructure, as described by the PBM. Finally, we propose a simplified scheme for our target experimental setup. It was found that the proposed model is capable of reproducing experimentally observed rheological behavior.

## 2. Model

The proposed model was built from a bottom-up perspective. That is, the objective is to reflect the fact that microstructural phenomena are responsible for the emergence of the macroscopically observed rheological properties. As suggested before, the steppingstone is the population balance equation, which in its most general form reads [28]:

(1) $$\begin{array}{c} \frac{\partial n}{\partial t}+\nabla_{\tilde{r}}\cdot \left(v_{\tilde{r}}n\right)+\nabla_{\tilde{x}}\cdot \left(v_{\tilde{x}}n\right)=\hat{h} \end{array}$$

The first term on the left-hand side of Equation (1) is an accumulation term of the cluster number density, *n*. The second and third terms quantify the change in number density due to advection in external and internal coordinates, respectively. The external coordinate set, $\tilde{r,}$ corresponds to physical space, while the internal set, $\tilde{x},$ corresponds to the tracked property or group of properties (e.g., cluster size). The term on the right-hand side of Equation (1) represents the net generation of clusters due to discrete processes.

Equation (1) describes in a generic way the redistribution of a particle population in time. However, the crux of the framework is the detailed description of birth and death of clusters, which are caused by the physical particularities of each system. If the system can be assumed to be well mixed, Equation (1) can be rewritten [28,29,30]:

(2) $$\frac{\partial n}{\partial t}=\frac{1}{2}\int_{0}^{x}\beta \left(x-\epsilon ,\epsilon ,t\right)n\left(x-\epsilon ,t\right)n\left(\epsilon ,t\right)d\epsilon -\int_{0}^{\infty }\beta \left(x,\epsilon ,t\right)n\left(\epsilon ,t\right)d\epsilon \;+\int_{0}^{\infty }b\left(x,\epsilon \right)S\left(\epsilon \right)n\left(\epsilon ,t\right)d\epsilon -S\left(x\right)n\left(x,t\right)\;$$

In the previous expression, the first two terms on the right-hand correspond respectively to the birth and death of clusters due to aggregation. The last two terms represent respectively the birth and death of clusters caused by breakage. The aggregation kernel, β, represents the rate at which aggregation events between particles of different size occur, while the selection function, S, indicates the rate at which clusters of size ε are selected to break. The breakage function, b, corresponds to a probability density function for the formation of clusters of size x after aggregates of size ε have broken up.

Equation (2) is a partial integro-differential equation. Due to its complexity, numerical techniques must be used when a solution under realistic conditions is desired. Although several alternatives exist, sectional methods, which rely on the discretization of the continuous CSD, are often preferred. The reason for this is their ability to accurately predict selected moments of the distribution, while giving acceptable results for the complete CSD and keeping computational demands at a satisfactory level [31]. In this case, the Cell Average Technique (CAT), developed by Kumar and associates [30,32] was implemented.

As suggested above, the Cell Average Technique approximates a continuous CSD into a finite number of size intervals (cells) that conform a grid. This transforms Equation (2) into a set of ordinary differential equations that can be solved by a higher order time integrator. CAT is capable of predicting the evolution of the cluster distribution in a more accurate way than other sectional methods and can be applied to simultaneous aggregation and breakage scenarios, as well as to other coupled problems [30,32]. The discretized formulation has the following form:

(3) $$\frac{dN_{i}}{dt}=B_{agg+break,i}^{CA}\;-D_{agg+break,i}$$

Equation (3) implies an ordinary differential equation for each cell in the grid. Here, N is the number concentration of clusters, which can be obtained by approximating the continuous number density in terms of Dirac-delta distributions:

(4) $$n\left(t,x\right)\approx \overset{I}{\underset{i=1}{\sum }}N_{i}\delta \left(x-x_{i}\right)$$

where I is the total number of cells in the grid.

The terms on the right-hand side of Equation (3) correspond respectively to birth and death of clusters in the i^th^ cell due to the combination of aggregation and breakage. The CA superscript denotes the term as being cell average modified.

In order to maintain consistency with the first two moments of the CSD, the cell average modified birth term is calculated as follows:

(5) $$B_{agg+break,i}{}^{CA}\;=B_{agg+break,i-1}\lambda_{i}{}^{-}\left({\bar{v}}_{i-1}\right)H\left({\bar{v}}_{i-1}-x_{i-1}\right)+\;B_{agg+break,i}\lambda_{i}{}^{-}\left({\bar{v}}_{i}\right)H\left(x_{i}-{\bar{v}}_{i}\right)+\;B_{agg+break,i}\lambda_{i}{}^{+}\left({\bar{v}}_{i}\right)H\left({\bar{v}}_{i}-x_{i}\right)+B_{agg+break,i+1}\lambda_{i+1}{}^{+}\left({\bar{v}}_{i+1}\right)H\left(x_{i+1}-{\bar{v}}_{i+1}\right)$$

Here, H is the Heaviside step function, while the function λ is defined as follows:

(6) $$\lambda_{i}{}^{\pm }\left(x\right)=\frac{x-x_{i\pm 1}}{x_{i}-x_{i\pm 1}}$$

The average volume of clusters formed in cell i, ${\bar{v}}_{i}$, can be computed using Equation (7).

(7) $${\bar{v}}_{i}\;=\frac{V_{agg,i}+V_{break,i}}{B_{agg,i}+B_{break,i}}$$

The net flux of volume into cell i, V_i_, due to aggregation and breakage are given by the following two expressions.

(8) $$V_{agg,i}=\overset{j\geq k}{\underset{\begin{array}{c} j,k\\ x_{i-\frac{1}{2}}\leq \left(x_{j}+x_{k}\right)<x_{i+\frac{1}{2}} \end{array}}{\sum }}\left(1-\frac{1}{2}\delta_{j,k}\right)\beta_{j,k}N_{j}N_{k}\left(x_{j}+x_{k}\right)$$

(9) $$V_{break,i}=\underset{k\geq i}{\sum }N_{k}S_{k}\int_{x_{i-\frac{1}{2}}}^{p_{k}^{i}}xb\left(x,x_{k}\right)dx$$

where:

(10) $$p_{k}^{i}=\{\begin{array}{c} x_{i}\;\;\;\;\;\;\;\;\;\;\;\;\;\;\;\;\;\;\;\;\;\mathrm{if}\;k=1\\ x_{i+\frac{1}{2}\;\;\;\;\;\;\;\;\;\;\;\;\;\;\;\;}\mathrm{Otherwise} \end{array}$$

The net rate of addition of clusters to cell *i* due to aggregation and breakage were computed with Equations (11) and (12).

(11) $$B_{agg,i}=\overset{j\geq k}{\underset{\begin{array}{c} j,k\\ x_{i-\frac{1}{2}}\leq \left(x_{j}+x_{k}\right)<x_{i+\frac{1}{2}} \end{array}}{\sum }}\left(1-\frac{1}{2}\delta_{j,k}\right)\beta_{j,k}N_{j}N_{k}$$

(12) $$B_{break,i}=\underset{k\geq i}{\sum }N_{k}S_{k}\int_{x_{i-\frac{1}{2}}}^{p_{k}^{i}}b\left(x,x_{k}\right)dx$$

The total birth rate at each cell is obtained from:

(13) $$B_{agg+break,i}=B_{agg,i}+B_{break,i}$$

The death term in Equation (3) is defined by:

(14) $$D_{agg+break,i}=D_{agg,i}+D_{break,i}$$

where:

(15) $$D_{agg,i}=N_{i}\overset{I}{\underset{k=1}{\sum }}\beta_{i,k}N_{k}$$

(16) $$D_{break,i}=S_{i}N_{i}$$

To solve the systems of Ordinary Differential Equations (ODEs) described by Equations (3) to (16), three additional closures are needed. Those are the aggregation kernel, selection function and breakage function. These expressions allow the model to cope with the specificities of the system, including hydrodynamics, size dependent interactions forces, etc.

The aggregation process is usually understood to take place in two steps: clusters are transported and collide with each other, and subsequently, they may become attached depending on the various interaction forces present [33]. These steps are usually described in the aggregation kernel in the form of a collision frequency term and an aggregation efficiency term. The former gives the rate at which clusters collide with each other, while the latter describes the probability of a collision producing an aggregation event. Thus, the aggregation kernel is given the following general form.

(17) $$\beta_{j,k}=\theta_{j,k}\lambda_{j,k}$$

where θ is the collision rate between clusters of size j and k, while λ is their aggregation efficiency.

Modelling the collision frequency involves understanding the mechanisms that promote collisions. In the case of concentrated suspensions, it is usually considered that collisions occur due to three main mechanisms: (1) Shear (orthokinetic mechanism), (2) Brownian motion (perkinetic mechanism) and (3) differential sedimentation [33,34,35,36]. The first mechanism refers to aggregates coming together due to gradients in the fluid velocity. Brownian motion produces collisions as a result of the temperature-dependent diffusion it causes. The final mechanism, differential sedimentation, represents collisions caused by the differences in sedimentation velocity that arise due to different cluster sizes.

**Figure 1:**
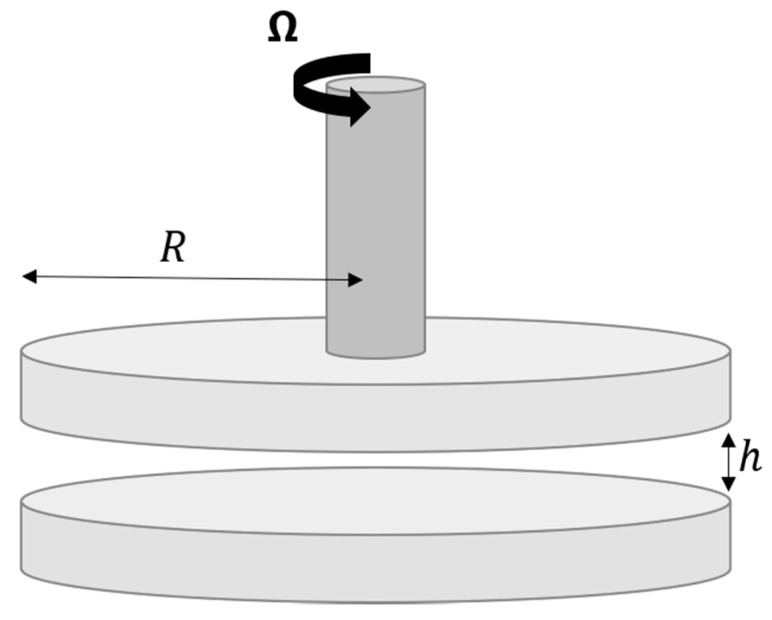
Geometry of the target experimental set-up of the proposed model. Two plates with radius R are separated by a gap h. The top plate rotates with an angular velocity Ω. Adapted from [41].

For the system at hand, perkinetic induced collisions were not considered because it has been observed that Brownian motion is not significant by virtue of the typical size range of cement clusters [2,8,35,37]. As a first approach, the model developed in this work is aimed at describing the observed flow behavior in a typical simplified experimental setup: a parallel plate rheometer (Figure 1). Taking this into account, sedimentation induced collisions were not considered. Given the typical settling velocities observed in fresh cement pastes [38,39], the small gap width in this experimental geometry and the time frame the model aims to capture, significant amounts of sedimentation should not occur. Thus, the collision frequency term in Equation (17) adopts the form of the widely known expression for orthokinetic aggregation under laminar shear flow [35,40]:

(18) $$\theta_{j,k}=k_{0}\frac{4}{3}\dot{\gamma }{\left(r_{j}+r_{k}\right)}^{3}$$

where $\dot{\gamma }$ is the shear rate, $k_{0}$ is a prefactor, and *r* is the radius of colliding clusters of size j and k.

It is noteworthy that errors introduced by the series of rather restricting assumptions under which Equation (18) was developed (i.e., clusters are spherical and follow a strictly rectilinear approach) were corrected by the inclusion of an aggregation efficiency term and a fractal dimension. Moreover, as discussed by Mohtaschemi et al. [18], these discrepancies scale in a form similar to the original kernel and thus can be captured by the efficiency term. Consequently, the latter, as a first approximation, was taken to be an adjustable parameter.

Unlike aggregation, the current understanding of breakage is more limited, which introduces uncertainties in the functional form of both the selection and breakage functions. For this reason, the semi-empirical expression presented by Puisto et al. [26], which aims to capture the competition between the hydrodynamic stress exerted on the clusters and the forces holding them together, was implemented. Therefore, the selection rate of aggregates of size k reads:

(19) $$S_{k}=S_{0}\dot{\gamma }\exp\left(-\frac{2F_{c}}{5\pi r_{k}^{2}\eta \dot{\gamma }}\right)$$

Here, $F_{c}$ is an effective bound force, η is the viscosity of the suspension, r is radius of the breaking cluster, and S_0_ is an efficiency term analogous to the one present in Equation (17).

Under the PBM framework, the modelling of the breakage frequency is done via probabilistic arguments, since this phenomenon is often considered to be random. Therefore, the breakage function first introduced in Equation (2) is a transition probability density function [28,29]. It is believed that the main breakage mechanism for cement aggregates is fracture, but other phenomena such as attrition can occur as well [42,43] Considering this and that no definitive evidence exists on the exact way in which breakup occurs, it was assumed that clusters are equally likely to form a pair of child agglomerates of any smaller size. This gives the breakage function the following form.

(20) $$b\left(x,x_{k}\right)=\frac{2}{x_{k}}$$

During the construction of population balance models, it is frequently assumed that particles are spherical. This simplification can be adequate in some cases (e.g., in bubbly flow applications), and it allows for the straightforward use of a sphere-equivalent radius in the calculation of aggregation and breakage rates. However, in the case of cement suspensions, aggregates adopt an irregular, porous structure. This has a large implication for the resulting rheological behavior, since the incorporated fluid increases the enclosed volume and the roughness of the clusters changes the way they interact with each other and the surrounding fluid [22,44]. Subsequently, fractal geometry [17,18,44,45,46,47] was used to describe the structure of clusters. The scaling relation between the mass of the aggregate and a characteristic length (in this case, its radius) is controlled by the mass fractal dimension, D_f_:

(21) $$M\;\propto l^{D_{f}}$$

The fractal dimension is a useful metric to assess the structural nuances of the clusters and its value varies from 1 (representing a linear aggregate) to 3 (for a perfectly spherical one). Thereupon, an effective volume fraction was calculated using this parameter with the objective of capturing the fact that the specific structure of non-spherical granules changes the way in which particles occupy volume in the suspension; affecting flow behavior [22].

(22) $$\phi_{eff}=\phi {\left(\frac{{\bar{r}}_{agg}}{{\bar{r}}_{p}}\right)}^{3-D_{f}}$$

where ${\bar{r}}_{agg}$ is the volume-weighted mean cluster radius given by PBM at any given time, while ${\bar{r}}_{p}$ is their initial size. Here, ϕ is the actual volume fraction of solids, which can be related to the more frequently used water to cement ratio (W/C) by:

(23) $$\phi ={\left(1+\frac{\rho_{p}}{\rho_{w}}\frac{W}{C}\;\;\right)}^{-1}$$

where $\rho_{p}$ and $\rho_{w}$ are the densities of clusters and water, respectively.

Finally, viscosity was calculated using the widely known Quemada constitutive equation [48]:

(24) $$\eta =\eta_{0}{\left(1-\frac{\phi_{eff}}{\phi_{max}}\right)}^{-\kappa }$$

where $\eta_{0}$ is the viscosity of the suspending medium, and $\phi_{max}$ represents the maximum packing fraction.

The model described by Equations (3) to (24) constitutes a system of ordinary differential equations, which requires an initial condition corresponding to the number density of clusters for each size class. The numerical solution of the system was conducted via a real-valued variable-coefficient ordinary differential equation solver based on the implicit Adams method [49]. An in-house Python code was used to implement the solver and other relevant subroutines.

As mentioned before, the present model is being built with the initial goal of reproducing rheological experiments currently being conducted in a parallel plate rheometer geometry. The objective being gaining a better understanding of the relationship between the results of those experiments and the structural evolution of cement suspensions. For this reason, through this document we use shear rate as the main engineering quantity. This variable appears explicitly in the model and therefore was deemed preferable to other parameters such as shear stress. Unless otherwise stated, it is assumed that the shear rate corresponds to the one attained at the edge of the plates, where it reaches a maximum, in accordance with Equation (25) [50].

(25) $$\dot{\gamma }\left(\hat{r}\right)=\frac{Ω\;\hat{r}}{h}$$

where $\hat{r}$ is the radial position, Ω is the angular velocity of the rotating plate, and h is the gap between plates.

The chosen rheometer geometry was selected for the reason that it is typically used in the field [41], and it is part of the experimental setup currently being used by the authors to conduct experimental studies whose results are pending publication. Other common geometries (such as the concentric cylinders configuration) can be incorporated into the model after replacing the previous equation with a suitable shear profile.

## 3. Results and Discussion

### 3.1. Grid Independence

The discretization method used by the model requires the segmentation of a continuous cluster size space into a number of size classes. This exercise inherently introduces errors in the calculation of relevant variables. For this reason, an investigation of the influence of the number cells was performed as a first step. Table 1 shows the parameters used. The size range and sample volume were chosen to represent typical values, while a uniform polydispersion was used as the initial condition in order to guarantee equivalence among simulations with a different number of classes.

**Figure 2:**
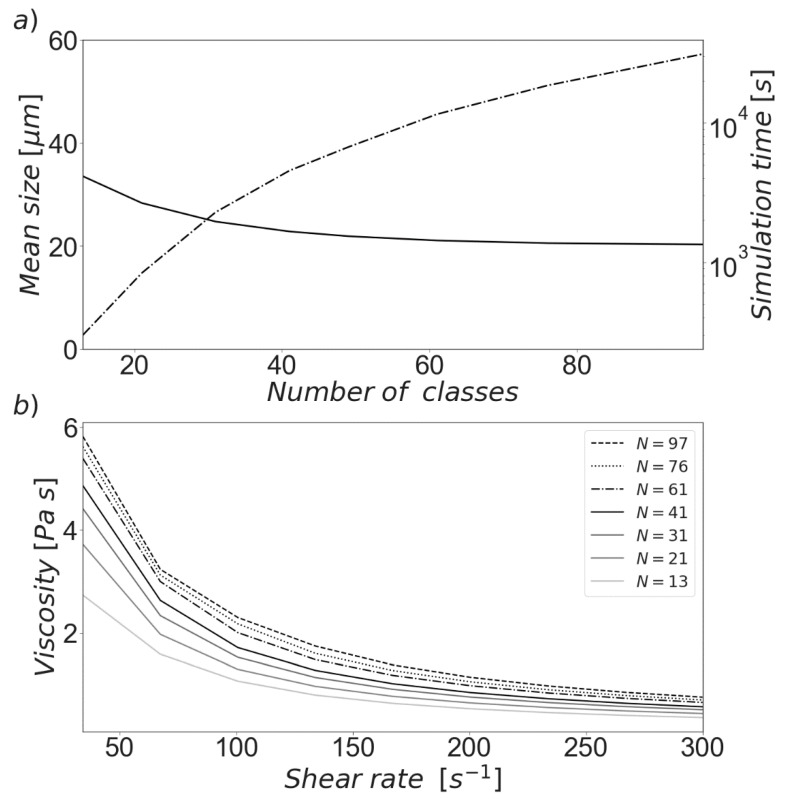
Effect of the number of size classes on relevant variables. (**a**) Mean cluster size (solid line) vs total simulation time (dash-dotted line) with a shear rate of 300 s^−1^. (**b**) Viscosity as a function of shear rate for different number of size classes.

After solving the presented system of ODEs, the results showed by Figure 2 were obtained. It is not surprising that an increase in the amount of cells led to significantly larger runtimes, as shown in Figure 2a. This is caused by the fact that each additional class implies an additional differential equation to be solved, with the accompanying calculations of agglomeration and breakage rates and of rheological information. The aforementioned figure also shows the dependence of the CSD (here represented as the mean size) calculated at a constant shear rate of 300 s^−1^. It was observed that the size distribution tends to reach a grid-independent state after a sufficiently large number of classes was used. In this case, simulations with 61 or more cells did not show significant variations in the resulting size distribution.

A similar situation occurs in the case of apparent viscosity. Figure 2b illustrates the influence of the number of classes over a wide range of shear rates. While a small systematic variation can be discerned, the calculated value of the variable also tends to stabilize when the grid is sufficiently fine. It is therefore necessary to find an appropriate balance between computational costs and accuracy, especially when the model is to be used in conjunction with experimentally derived data.

### 3.2. Sensitivity Analysis

One second element that must be considered in order to assess the applicability of the proposed model is the sensitivity of the output to changes in the numerical values of relevant variables. This was achieved with a global sensitivity analysis based on the Sobol method [51,52]. Global analysis is useful to distinguish the individual contribution of each parameter as well as the interactions among them, over the entire parameter range, which must be determined a priori. Considering that the presence of these interactions can be significant in highly non-linear models, it is important to examine them and not only the first order effects. The downside lies in the high associated computational costs.

The selected technique is able to decompose the variance of the model output into a series of summands of the variance of the input parameters. With this, it is possible to determine how much the variability in the model output can be attributed to individual parameters or interactions among them. With the objective of producing meaningful estimates, the parameter space must be thoroughly scanned via Monte Carlo methods, which implies a large number of simulation runs, hence the large computational effort previously mentioned [51,52].

Table 2 shows the basic parameters used to conduct the sensitivity analysis. The experimental CSD and W/C data for cement pastes presented by Ferron et al. [42] was used as a starting point. Four main parameters were studied, namely, aggregation and breakage efficiencies, effective cluster bound force and fractal dimension. The efficiencies were varied between 0 (no aggregation or breakage) and 1 (all aggregation or breakage events are successful). The work of Flatt [53] was used to estimate the range of the effective bound force for cement suspensions. In turn, the fractal dimension was varied between 1 (linear aggregates) and 3 (spherical aggregates). Apparent viscosity was chosen as the output variable and simulations were conducted for a single, constant shear rate in order to reduce the computational effort.

**Figure 3:**
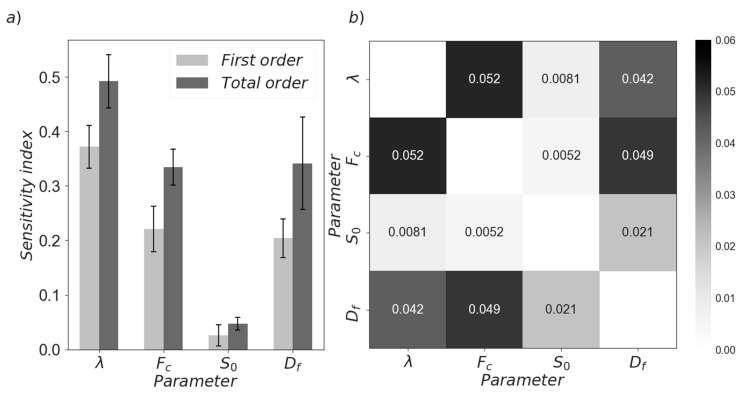
Sensitivity of the model to its parameters. (**a**) Total and first order effects. (**b**) Second order effects.

Figure 3a shows the first order and global sensitivity indices obtained from the analysis. The former indicates the individual contribution of each parameter to the output variance, while the latter is an aggregation of the individual contributions plus all the interaction effects. Figure 3b illustrates the various second order interactions among the studied variables. With the exception of breakage efficiency, it was found that the chosen model output is highly sensitive to all of the relevant parameters. This suggests that the present mathematical formulation is able to offer the required flexibility for its intended purpose. Furthermore, the significant differences between the total and first order indices, as well as the magnitudes of the calculated second order interactions indicates that the model is highly non-linear. As shown by Figure 3b, all parameters have more than one significant interaction with another variable, which indicates again that the variability of the calculated apparent viscosity is caused by complex series of intertwined effects and feedback loops. This situation is a necessary one, as rheological dynamics of cement suspensions are intricate and depend on several different phenomena. However, this can cause identifiability issues during calibration. Therefore, special care must be taken during the adjustment of parameters to experimental data.

Although sensitivity analysis is only concerned with the internal mathematical structure of a model, it can offer some insight regarding the ability of said model to represent reality. For instance, aggregation dynamics are the main driving force in the evolution of CSD during the initial time period after water addition. Consequently, it is adequate that the model is capable of responding to changes in this variable, as indicated by the high sensitivity index attributed to the aggregation efficiency. Moreover, the presence of several interparticle forces is of high importance for the observed rheological responses. In this perspective, the high sensitivity of the model to the former parameter as well as to the effective bound force—both of which are directly related to said forces—indicates that the current formulation can be used to capture the nuances introduced by these phenomena. Likewise, the model shows an ability to respond to changes in the fractal dimension, that is, to changes in the microstructure of the agglomerates. This, in conjunction with the relatively large magnitude of the second order effects, insinuates that this variable can be used to bridge the gap between microscopic and macroscopic phenomena and that it can be adequate to capture structural effects that depend on flow conditions [42].

It is noteworthy that it is not necessary that the aforementioned parameters are treated as fitted variables. While this approach can make the application of the model more straightforward and may reduce computational expenses, other strategies may be implemented to calculate one or more of said parameters. For example, the DLVO theory [54] could be implemented to approximate the values of the bound force and aggregation efficiency. However, lack of data regarding Hamaker constants and other vital parameters for cement clinker components may limit the added value obtained from this exercise [53]. In a similar manner, expressions for the fractal dimension such as the one developed by Selomulya et al. [55] can be included to add more depth to the microstructural side of the model, at the cost of having more fitted parameters. Moreover, the scaling theory of Gennes for polymer adsorption [56] and the DLVO approximation can be implemented to directly account for the effect of dosing, if more resolution is desired.

### 3.3. Predicted Rheological Responses

**Figure 4:**
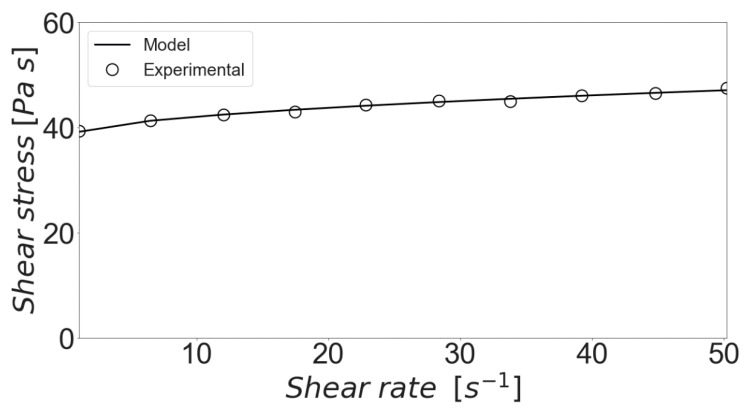
Experimental vs model shear stress profile.

Now that the numerical performance of the model has been addressed, it is important to discuss the rheological information that can be obtained from it. As an initial step, experimental data provided by Bentz et al. [16] was used to perform an adjustment of the model parameters with the quasi-Newton algorithm of Broyden, Fletcher, Goldfarb and Shanno [57]. The proposed model was used to reproduce the shear procedure performed by the authors and, as can be seen in Figure 4, a very good agreement can be obtained for the used experimental procedure and initial cluster size distribution. The fitted parameters were found to be within the ranges described above. More specifically, the aggregation and breakage efficiencies were fixed as 0.1498 and 0.8843, respectively. The bound force was assigned a value of 6.6501 nN, which is considered to be large for clusters experimenting colloidal forces [26,53]. This implies a high degree of resistance of the aggregates to shear forces. In turn, the fractal dimension assumed the relatively high value of 2.4338. In prior studies, it has been observed that shear induced aggregation tends to produce clusters with large fractal dimensions [26,58], indicating clusters of only a rough spherical shape.

As a second step and for demonstration purposes, a different shear procedure was simulated. The procedure was composed of a total of 30 discrete steps, consisting in an initial acceleration from 1 to 150 s^−1^ and a subsequent deceleration back to 1 s^−1^. The model parameters remained the same. It must be noted, however, that further calibration including more CSD data and a larger range of shear rates would be desirable for practical applications. The water to cement ratio was chosen to be 0.35.

**Figure 5:**
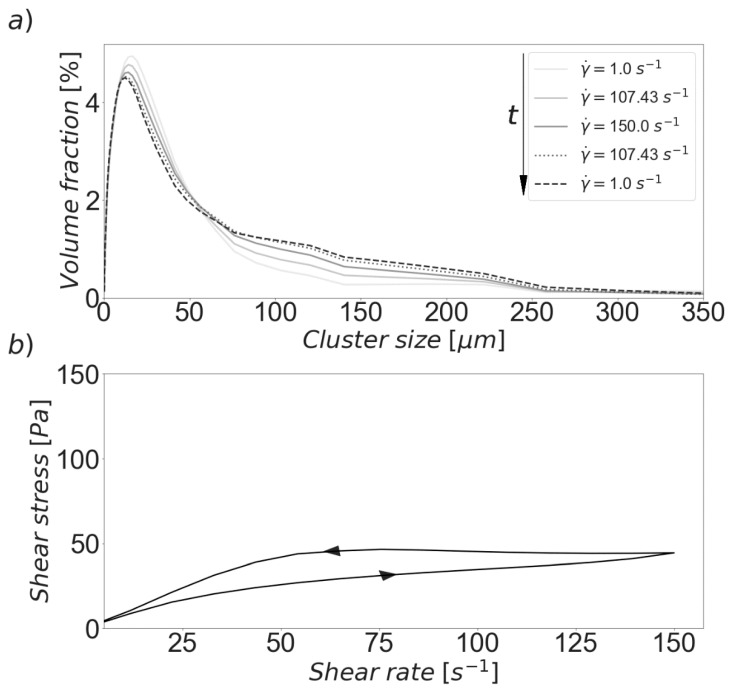
Results of simulated shear rate ramp and deceleration. The arrows indicate the time progression during the shear procedure. (**a**) Evolution of the CSD. (**b**) Hysteresis loop.

Figure 5a shows the calculated size distribution obtained after selected steps. It can be seen that the polydispersity of the CSD increased as the shear procedure evolved. This is caused by the agglomeration of the smaller clusters, while the largest size classes tended to breakdown to produce smaller aggregates. The net result is an overall increase in the mean size during the simulated experiment. Furthermore, it can be observed that size distribution obtained for a given shear rate during the acceleration part of the procedure is not equal to the one calculated for the same shear rate during the deceleration period. For example, it can be seen that the CSD displayed in Figure 5a for a shear rate of 107 s^−1^ during the acceleration period (solid line) is significantly different to the distribution of the paste when shear returned to the same value in the second part of the procedure (dotted line). As such, this means that the model is capable of reproducing the inherently transient phenomena that governs the structural build-up and breakdown experienced by cement pastes in the simulated type of rheological experiment.

The simulated rheological behavior is illustrated by Figure 5b. It was assumed that the stress response is viscous. The magnitudes of the stress are generally smaller than those presented in Figure 4 for the reason that the pre-shearing operations performed during the original experiments were not replicated in the simulation being described. Nonetheless, it is notable that the resulting hysteresis loop is comparable to experimentally derived ones reported in the literature for similar conditions [41,59]. It can be observed that the deceleration ramp appears above the acceleration ramp, which signifies that the suspension is displaying anti-thixotropic behavior. That is, during each step, agglomeration is dominant over breakdown, causing the clusters to grow faster than they can be dispersed due to shear forces, and thus, the net is result is the production of increasing shear stresses. These dynamics are caused by the presence of different competing mechanisms. For instance, the non-sphericity of the clusters facilitates aggregation but makes them more vulnerable to breakup due to shear forces [58].

**Figure 6:**
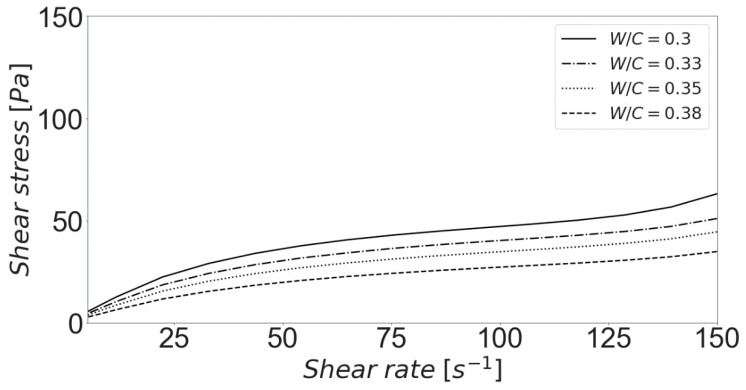
Influence of the water to cement ratio (W/C) on the predicted flow curves.

As mentioned before, the solids volume fraction (or, equivalently, the water to cement ratio) is a key variable for the resulting rheology of a given cement suspension. Subsequently, this parameter is a critical during formulation and for the later performance of the material. Figure 6 shows the predicted flow curves for different water to cement ratios. Only the acceleration ramp of the previously described shear procedure was simulated. The same set of parameters and initial experimental conditions were used. As the water content diminishes, higher shear stresses can be observed. The growing jamming of the aggregates makes flow increasingly more difficult, resulting in the described macroscopic response. Previous experimental studies [60,61,62] are consistent with model results under comparable conditions.

The fact that the proposed model is capable of keeping track of the transient evolution of the suspension microstructure allows it to natively reproduce complex macroscopic responses, such as the ones discussed before. This feature permits greater flexibility in the prediction of rheology, since complex phenomena can be considered in a direct manner. As stated before, this is of importance because the evolution of the cluster size and its degree of polydispersity are critical for the determination of macroscopic flow. These dynamics cannot usually be captured by using volume fraction information alone.

### 3.4. Spatially Resolved Model

Finally, the feasibility of using the model to offer insight into the internal dynamics of its target experimental setup was investigated. In classical rheometry, it is usually assumed that the velocity field of a fluid inside a parallel plates geometry only has one linear azimuthal component, which allows for the derivation of Equation (25) [50]. However, the presence of a secondary radial component has long been identified as a possible source of significant errors, especially when samples are complex suspensions [50,63,64]. Therefore, it is very important to guarantee that this secondary flow is not of significance. This can be very challenging in the case of cement pastes, since their opacity limits the applicability of most experimental velocimetry techniques. The use of modelling methods after they have been adequately validated can thus help to alleviate this problem.

Keeping this in mind, a simple spatially resolved model was developed. The classical analytical solution of Savins and Metzner was used [63]. Assuming a low Reynolds number, the radial velocity component of a fluid under torsional flow is given by Equation (26).

(26) $$v_{r}=\frac{\rho {Ω}^{2}h^{2}\hat{r}F\left(\hat{z},h\right)}{12\eta }$$

where ρ is the density of the suspension, and $\hat{z}$ is the vertical position. The function F arises from the integration of the momentum equation. Its definition is given in the original reference [63]. The primary velocity component remains unchanged and it reads:

(27) $$v_{\theta }=\frac{Ω\hat{r}\hat{z}}{h}$$

**Figure 7:**
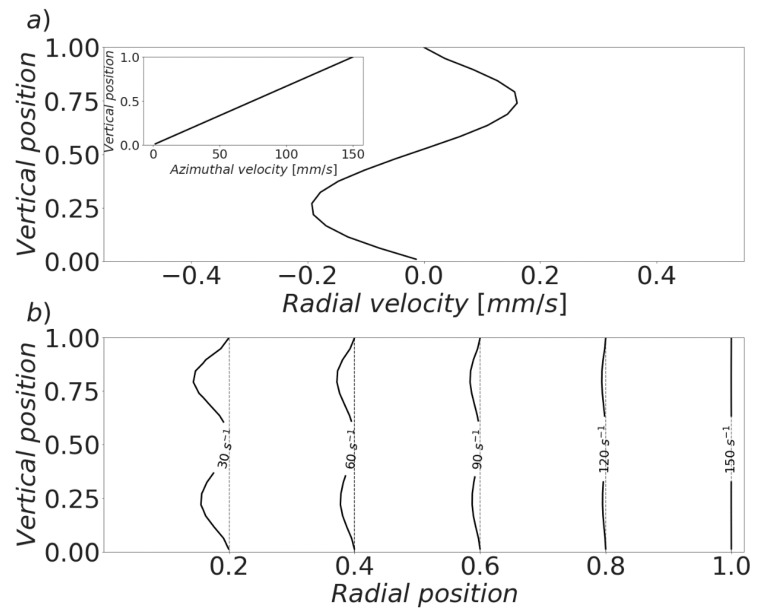
Local velocity and shear rate profiles in a parallel plates geometry. The rotating plate is located at the top. (**a**) Primary (azimuthal, inset) and secondary (radial) velocity profiles at the edge of the geometry. (**b**) Shear rate profile. The center of the geometry is located at the left. The vertical dashed lines correspond to the ideal design scenario (no secondary flow).

**Figure 8:**
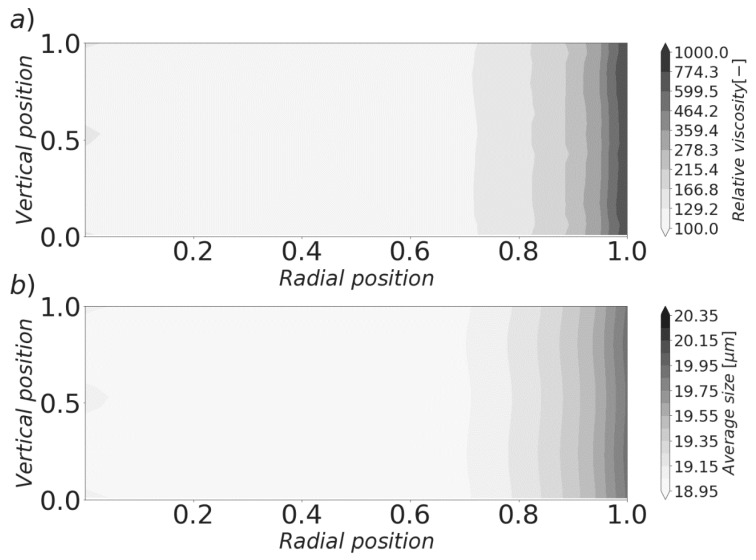
Local viscosity and average size profiles in a parallel-plates geometry. The rotating plate is located at the top. The center of the geometry is located on the left. (**a**) Relative viscosity profile. (**b**) Average size profile.

Equations (26) and (27) allow for a more comprehensive picture of the shear rate profile inside the experimental apparatus to be constructed. The shear rate becomes a function of the suspension viscosity, which generates a double way coupling in the mathematical structure of the model. After solving it at 400 discrete positions, the results showed by Figure 7 and Figure 8 were obtained. The same set of parameters previously described were used. Simulations were performed under a constant applied shear rate of 150 s^−1^. That is, the angular velocity of the rotating plate was kept in such a way that the shear rate at the edge of the plates attained that value. The gap was taken to be 1 mm, while the plates diameter assumed a value of 40 mm.

The magnitude of the velocity of the secondary flow at the edge of the disks, under the studied conditions, can be observed in Figure 7a. The shape of this profile is nontrivial, as it depends on a complex set of interactions. Although this strikes a contrast with the simplicity of the radial velocity component, the magnitude of the latter is significantly larger than the magnitude of the secondary flow. The small gap and high viscosity of the fluid limit inertial effects, producing a practically unperturbed circumferential motion. The net result is a small, albeit noticeable, deviation of the shear rate profile from the ideal one. The dashed vertical lines displayed in Figure 7b correspond to the usually assumed shear rate profile in parallel disks rheometers. In accordance with Equation (25), it is commonly assumed that shear evolves linearly with radius and that it is constant along the gap of the geometry. However, the presence of a secondary flow causes a divergence from the expected profile. This deviation is more significant near the center of the apparatus, where the magnitudes of both velocities are closer. While this effect seems small in this case, it may start to gain more significance when the gap and/or shear rate are increased. A larger water to cement ratio is also expected to produce more significant secondary flows.

The resultant relative viscosity (defined as the ratio between the suspension apparent viscosity and the viscosity of water) profile is displayed in Figure 8a. The simulated paste exhibited a shear-thickening behavior, which is consistent with experimental studies that have found that cement suspensions tend to behave in this way when the water to cement ratio is below a value situated around 0.4 [62]. The radially dependent changes in this variable can thus be attributed to the primary evolution of the shear rate in the geometry: The larger shear forces experimented by the suspension far from the center increase the aggregation rate, which produces a higher number of large sized aggregates, hampering flow. This phenomenon can be more easily appreciated in Figure 8b, which shows that the cluster size distribution tends to shift towards bigger sizes as the position approaches the edge of the geometry. The presence of the secondary flow introduced small vertical dependences to both viscosity and average size. This is caused by the variability in aggregation and breakage rates caused by the small variances in the shear rate.

The limitations of the chosen analytical profile can introduce constraints in the predictive ability of the model. However, after experimental validation, it could play a useful role as an auxiliary to experimental studies. Furthermore, more sophisticated tools, such as computational fluid dynamics, could be used to generate more detailed velocity profiles that can then be coupled with the PBM based model.

## 4. Conclusions

The population balance framework was identified as a valuable tool in the study of the rheology of cement suspensions during the time period immediately following water addition. The model proposed here is capable of relating the evolution of the microstructure with the macroscopic behavior of the flow. It is believed that this capability is fundamental in the development of models with the flexibility required to tackle the several process and environmental conditions usually found in cement technology. Therefore, the ability of PBM to transiently evolve a cluster size distribution given said conditions allows it to reproduce complex rheological behavior that cannot be usually predicted with simpler models. In principle, the applicability of the model can be extended to concrete suspensions by adding perkinetic and differential sedimentation expressions to the aggregation kernel.

The cell average technique was implemented to numerically solve the population balance equation. Considering that this entails the discretization of the cluster size space, the errors associated with the number of size classes were investigated. While some systematic shifts in the values of predicted viscosity were observed, an essentially grid independent state can be reached after an adequately high number of cells is used. In a similar manner, Monte Carlo methods were implemented to test the sensitivity of the model output to its main set of parameters. Although the presence of significant second order interactions was pinpointed as a possible source of identifiability issues during experimental calibration, it was found that the chosen group of model parameters can offer the flexibility required to reproduce the effects of the multiple competing phenomena usually governing rheology in cement pastes.

The model was found to be able to reproduce the complex rheological characteristics usually found in cement suspensions. The anti-thixotropic behavior emerging from the simulation of a typical shear procedure is well in line with experimental observations. These features are caused by the transient evolution of the cluster size distribution during the procedure. As it evolves, the aggregation mechanisms tend to dominate over breakage, which leads to increasing stress values. Even though additional experimental calibration and validation is required, the model can be used to further the knowledge of the multiscale set of interactions producing macroscopic observables in fresh pastes. The use of modelling tools is of special importance due to the fact that the high opacity and concentration in typical cement pastes complicates enormously the application of optical experimental techniques.

An analytical solution for torsional flow was used to generate a more complete description of the shear field inside the target experimental geometry. Subsequently, the model was spatially resolved to study the effect of secondary flows when the coupling with the CSD evolution is taken into account. The high viscosity of the suspension as well as the small gap between plates limit inertial effects and thus the influence of secondary flows. These are, however, noticeable in the simulated results. Further study of potential errors caused by these phenomena may therefore be of interest. In addition, the shear-thickening behavior caused by the radial dependence of shear rate in a parallel disk geometry was predicted by the spatially resolved model. As in previous cases, this is produced by the adjustment of the microstructure to its local environment.

## Figures and Tables

**Table 1:** Grid independence test parameters.

Parameter	Value
Initial condition	Uniform polydispersion
Size range [µm]	1–100
Total volume [m^3^]	1 × 10^−5^
Type of grid	Uniform
Number of size classes	13–97
Simulation type	Shear rate sweep (30–300 s^−1^)
Simulation hardware	Intel i7 6700k 4.0 GHz, 16GB RAM

**Table 2:** Sensitivity analysis parameters.

Parameter	Value
Initial condition	Experimental [42]
Size range [µm]	1–100
Total volume [m^3^]	1 × 10^−5^
Type of grid	Non-uniform
Studied parameters	Aggregation efficiency (λ), effective bound force (Fc), breakage efficiency (S0), fractal dimension (Df)
Studied output	Viscosity (η)
Simulation type	Constant shear rate (100 s^−1^)
Number of samples	20000
